# Amino Acid Availability Determines Plant Immune Homeostasis in the Rhizosphere Microbiome

**DOI:** 10.1128/mbio.03424-22

**Published:** 2023-02-14

**Authors:** Yang Liu, Andrew J. Wilson, Jiatong Han, Alisa Hui, Lucy O’Sullivan, Tao Huan, Cara H. Haney

**Affiliations:** a Department of Microbiology and Immunology, The University of British Columbia, Vancouver, Canada; b Department of Chemistry, The University of British Columbia, Vancouver, Canada; c Department of Molecular Biology, Massachusetts General Hospital, Boston, Massachusetts, USA; University of Toronto

**Keywords:** PTI, amino acid biosynthesis, innate immunity, pH, plant-microbe interactions, rhizosphere-inhabiting microbes

## Abstract

Microbes possess conserved microbe-associated molecular patterns (MAMPs) that are recognized by plant receptors to induce pattern-triggered immunity (PTI). Despite containing the same MAMPs as pathogens, commensals thrive in the plant rhizosphere microbiome, indicating they must suppress or evade host immunity. Previous work found that bacterial-secreted gluconic acid is sufficient to suppress PTI. Here, we show that gluconic acid biosynthesis is not necessary for immunity suppression by the beneficial bacterial strain Pseudomonas simiae WCS417. We performed a forward genetic screen with EMS-mutagenized *P. simiae* WCS417 and a flagellin-inducible *CYP71A12_pro_:GUS* reporter as a PTI readout. We identified a loss of function mutant in ornithine carbamoyltransferase *argF*, which is required for ornithine conversion to arginine, that cannot suppress PTI or acidify the rhizosphere. Fungal pathogens use alkalization through production of ammonia and glutamate, and arginine biosynthetic precursors, to promote their own growth and virulence. While a *ΔargF* mutant has a growth defect in the rhizosphere, we found that restoring growth with exogenous arginine resulted in rhizosphere alkalization in a mutant that cannot make gluconic acid, indicating that arginine biosynthesis is required for both growth and acidification. Furthermore, blocking bacterial arginine, glutamine, or proline biosynthesis through genetic mutations or feedback inhibition by adding corresponding amino acids, resulted in rhizosphere alkalization. Untargeted metabolomics determined that ornithine, an alkaline molecule, accumulates under conditions associated with rhizosphere alkalization. Our findings show that bacterial amino acid biosynthesis contributes to acidification by preventing accumulation of ornithine and the resulting alkalization.

## INTRODUCTION

A myriad of microorganisms, including pathogens and mutualists, live in the plant rhizosphere and actively influence plant fitness (1). To protect themselves from pathogens, plants use pattern recognition receptors (PRRs) that can specifically sense microbe-associated molecular patterns (MAMPs), which are evolutionarily conserved across diverse microbes. Perception of MAMPs results in pattern-triggered immunity (PTI), which includes a reactive oxygen species burst, calcium influx, and defense gene expression (2). As both pathogens and commensals contain MAMPs that can be recognized by the plant innate immune system, both must suppress or evade immunity in order to successfully colonize. While mechanisms of immunity suppression by pathogenic bacteria are well-established, how commensal microbes evade or suppress plant immunity to promote their own fitness is poorly understood.

Some successful pathogens can suppress PTI by injecting effector proteins into the plant cytosol via the type III secretion systems (T3SS) (3). In addition to injecting effectors, pathogenic bacteria can manipulate phytohormones to suppress host immunity. The pathogenic bacterium Pseudomonas syringae pv tomato DC3000 (*Pto*) can secrete the phytotoxin coronatine (COR) that mimics the active form of jasmonic acid (JA), JA-lIe, to promote JA-dependent defense. Since the JA and salicylic acid (SA) pathways antagonize each other, inducing JA signaling by COR suppresses SA signaling, which is critical for resistance against *Pto* (4). Lastly, instead of suppressing host immunity, pathogenic microbes can degrade their MAMPs to prevent recognition by PRRs. For instance, P. syringae can secrete AprA, an extracellular alkaline protease that can degrade flagellin monomers, thereby avoiding immune recognition (5).

Although commensals also have MAMPs that have the potential to induce PTI, many do not induce immune responses, suggesting that commensals can suppress or evade the plant immune system (6). A growth promoting and biocontrol bacterial strain, Pseudomonas sp. WCS365, can evade host immunity by fine tuning biofilm formation (7). Pseudomonas *capeferrum* WCS358 can suppress root local immunity by secreting organic acids to lower the pH of the rhizosphere (8). Dyella japonica suppresses root immunity through a Type II secretion-dependent mechanism without affecting rhizosphere pH (9). Collectively, these findings indicate that rhizosphere commensals possess diverse mechanisms to modulate host immunity.

The beneficial root-associated bacterial strain, P. simiae WCS417, was previously shown to suppress root immunity (8, 10). It lowers the pH of the rhizosphere to a greater extent than *P. capeferrum* WCS358, and produces more gluconic acid (8); however, we found that deletion of *pqqF*, which results in loss of gluconic acid biosynthesis and immunity suppression in *P. capeferrum* WCS358 (8), does not impair the ability of WCS417 to suppress rhizosphere immunity. Here, we describe a forward genetic screen that identified a novel mechanism of root immunity suppression in *P. simiae* WCS417, where amino acid biosynthesis prevents rhizosphere alkalization and suppresses immunity.

## RESULTS

### Acidification is sufficient, but not necessary, for *Arabidopsis* immunity suppression by *P. simiae* WCS417.

**FIG 1:**
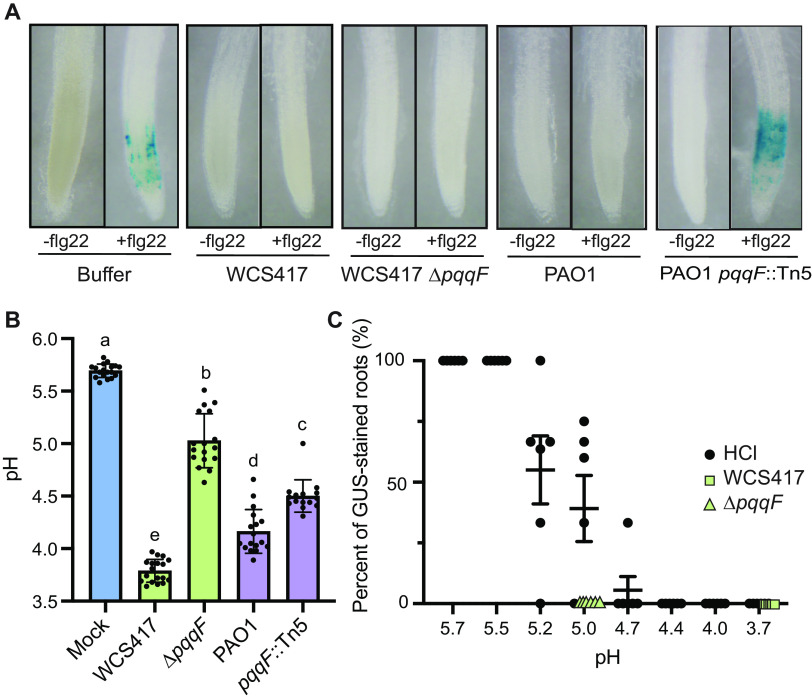
*pqqF* is not required for host immunity suppression in *P. simiae* WCS417. (A) While loss of function of P. aeruginosa PAO1 *pqqF* results in inability to suppress expression of *CYP71A12_pro_:GUS* expression, the *P. simiae* WCS417 *pqqF*-deficient mutant retains the ability to suppress flg22-induced *CYP71A12_pro_:GUS* expression. (B) Deletion or disruption of *pqqF* results in a pH increase in WCS417 and P. aeruginosa PAO1. Individual data are the result of a single well of seedling exudates. Statistics were determined by using one-way ANOVA and Tukey’s HSD. Error bars represent mean ± SD, and letters indicate differences at *P* < 0.05. (C) Percent of GUS-stained *CYP71A12_pro_:GUS* roots in the presence of flg22 under a pH gradient (black dots). pH of WCS417 (green squares) and the *pqqF* mutant fully suppress flg22-triggered *CYP71A12_pro_:GUS* expression. Each dot represents the percent of blue roots from 1 to 3 wells of a temporal experiment containing 3 to 4 roots per well. All experiments were independently repeated at least 3 times.

Gluconic acid biosynthesis via *pqqF* is necessary for *Arabidopsis* rhizosphere immunity suppression in *P. capeferrum* WCS358 and Pseudomonas aeruginosa PAO1 as measured by expression of a PTI-inducible reporter *CYP71A12_pro_:GUS* expression (8). *CYP71A12* is involved in biosynthesis of the antimicrobial camalexin and is induced in the root elongation zone or maturation zone upon sensing MAMPs such as flg22 or chitin (10). As a result, induction of *CYP71A12_pro_:GUS* reports the activation of PTI. *P. simiae* WCS417 produces more gluconic acid and lowers the pH of the rhizosphere to a greater extent than WCS358 suggesting WCS417 *pqqF* is also required for rhizosphere immunity suppression (8). Surprisingly, we found that the WCS417 *ΔpqqF* mutant can still completely suppress flg22-triggered expression of *CYP71A12_pro_:GUS* (Fig. 1A). We found that while *P. simiae* WCS417 acidifies seedling exudates to a pH of 3.7, a clean deletion of *pqqF* in *P. simiae* WCS417 resulted in a significant increase in the pH of seedling exudates to pH 5.0 (Fig. 1B). In contrast, while wildtype P. aeruginosa PAO1 lowers the pH of the seedling exudates to pH 4.0 and supresses *CYP71A12_pro_:GUS*, disruption of PAO1 *pqqF* resulted in a less dramatic increase in the pH of seedling exudates to pH 4.5 (Fig. 1B) but results in a complete loss of suppression in host immunity (Fig. 1A; (8)). Collectively these data suggest a gluconic acid independent mechanism of immunity suppression by *P. simiae* WCS417.

Previous work showed that lowering the rhizosphere pH to 3.7 with hydrochloric acid (HCl) resulted in complete inhibition of *CYP71A12_pro_:GUS* expression (8). Lowering the rhizosphere pH to between 5.5 to 4.6 resulted in partial immunity suppression, indicating that we should expect about 50% of roots to retain *CYP71A12_pro_:GUS* expression at pH 5.0, the pH of the *P. simiae* WCS417 *ΔpqqF* mutant growing in seedling exudates. We tested the effect of a pH gradient on suppression of *CYP71A12_pro_:GUS* and indeed found that modifying the pH of the rhizosphere to 4.7–5.0 in the absence of bacteria results in intermediate suppression of plant immunity, with about 50% of roots retaining *CYP71A12_pro_:GUS* expression (Fig. 1C). The pH of the WCS417 *ΔpqqF* mutant is around 5.0, but results in complete inhibition of *CYP71A12_pro_:GUS* expression (Fig. 1C). These data suggest that WCS417 possesses additional mechanisms to suppress host immunity.

### *P. simiae* WCS417 a*rgF* is required for rhizosphere acidification and immunity suppression.

**FIG 2:**
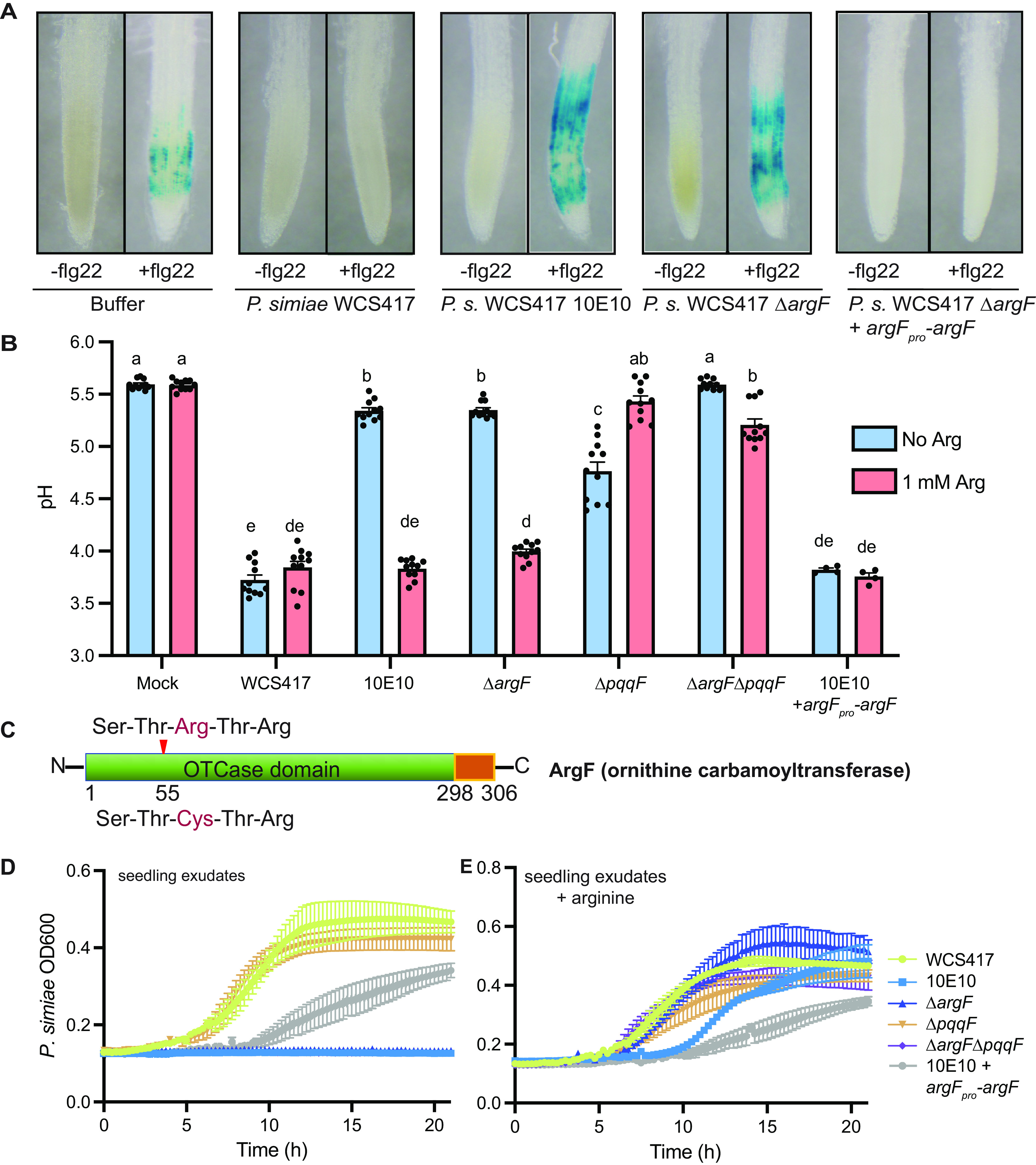
An EMS screen identified a mutant in *argF* that is required for both Pseudomonas growth and rhizosphere acidification. (A) A mutant, 10E10, was identified from an EMS screen for *P. simiae* mutants that cannot supress flg22-triggered induction of *CYP71A12_pro_:GUS* expression or (B) acidify the rhizosphere. An *ΔargF* mutant phenocopied the inability of the 10E10 mutant to suppress immunity suppression and acidification, and expression of *argF_pro_*-*argF* on a plasmid restored the immunity suppression and acidification of the 10E10 mutant. Addition of exogenous arginine lowered the pH of the *ΔargF* and 10E10 mutant, but resulted in alkalization of a Δ*pqqF* or a *ΔpqqFΔargF* double mutant growing in seedling exudates. All the experiments were independently repeated at least 3 times. Statistics were calculated by using one-way ANOVA and Tukey’s HSD. Error bars represent mean ± SD, and letters indicate differences at *P* < 0.05. (C) The mutation in the 10E10 mutant is within the catalytic site of *ΔargF*, and results in a predicted loss of function mutation. (D) The 10E10, the *ΔargF*, and the *ΔargFΔpqqF* mutants had a growth defect in the seedling exudates. (E) Growth of *ΔargF* and the *ΔargFΔpqqF* mutants were rescued by the addition of exogenous arginine.

To identify additional genes that are necessary for host immunity suppression, we generated an EMS-mutagenized library of *P. simiae* WCS417, and screened for mutants that were unable to suppress flg22-mediated induction of the *CYP71A12_pro_:GUS* reporter. We screened 960 EMS-mutagenized colonies of WCS417 in duplicate for their ability to suppress flg22-induced expression of the *CYP71A12_pro_:GUS* reporter. A single mutant, named 10E10, was found incapable of suppressing flg22-induced immunity (Fig. 2A). We found that 10E10 completely failed to reduce the pH of seedling exudates, suggesting that it might contribute to immunity suppression through rhizosphere acidification (Fig. 2B).

Because we only identified one mutant from the screen, we wondered if mutations in WCS417 that result in a loss of immunity suppression are rare, or if our screen was not saturating. To test this, we determined whether we could identify an allele of *pqqF* in our screen. Gluconic acid has previously been shown to be required for zinc solubilization, and so we tested whether we could identify a mutant unable to solubilize zinc. By growing bacteria on zinc phosphate media (11), only the strains that can solubilize zinc phosphate will produce a clear halo on the plate. We found a single mutant, 4E4, that cannot solubilize zinc phosphate (Fig. S1). The genome of 4E4 was sequenced, and we identified mutations in both *pqqF* and *pqqB* (Data Set S1, Table S1). However, 4E4 can still suppress flg22-triggered *CYP71A12_pro_:GUS* expression, which is consistent with our finding that the WCS417 *ΔpqqF* mutant can still suppress plant immunity. As our screen successfully identified a mutation in *pqqF*, which suggests that mutants that fail to suppress root immunity might be rare in *P. simiae* WCS417.

10.1128/mbio.03424-22.1 FIG S1 *P. simiae* WCS417 can solubilize ZnSO_4_, while the mutant 4E4 cannot. Rescreening the *P. simiae* WCS417 EMS library on media containing ZnSO_4_ and glucose identified a single mutant that could not solubilize zinc. Sequencing the 4E4 mutant identified point mutations in both *pqqF* and *pqqB*. The 7A1 strain can solubilize ZnSO4, and is shown as a positive control. Download FIG S1, PDF file, 0.5 MB. Copyright © 2023 Liu et al. 2023 Liu et al. https://creativecommons.org/licenses/by/4.0/ This content is distributed under the terms of the Creative Commons Attribution 4.0 International license.

10.1128/mbio.03424-22.12 DATA SET S1 The process of HILIC+/− shows results from metabolomics analysis. Data Set S1, Table S1, A total of 35 non-synonymous candidate genes from genome mapping of the 10E10 WCS417 mutant. Data Set S1, Table S2, total of 48 non-synonymous candidate genes from genome mapping of the 4E4 WCS417 mutant. Data Set S1, Table S3 Stains used in this study. Download Data Set S1, XLSX file, 0.5 MB. Copyright © 2023 Liu et al. 2023 Liu et al. https://creativecommons.org/licenses/by/4.0/ This content is distributed under the terms of the Creative Commons Attribution 4.0 International license.

We sequenced the genome of the *P. simiae* WCS417 10E10 mutant, and identified 35 non-synonymous mutations with respect to the parental WCS417 strain (Data Set S1, Table S2). To narrow down candidate genes, we made use of a PAO1 transposon insertion library (12), and tested transposon insertion mutants in PAO1 orthologs of genes from the mapping list that we hypothesized were most likely to contribute to immunity suppression (Fig. S2). We found that an insertion in *argF* uniquely impaired the ability of PAO1 to acidify seedling exudates (Fig. S2). *ArgF* encodes ornithine carbamoyltransferase, which is involved in arginine biosynthesis, by converting l-ornithine to L-citrulline (13, 14). The mutation in *argF* in *P. simiae* WCS417 is predicted to affect its catalytic site Ser-Thr-Arg-Thr-Arg, where a C to T change is predicted to convert the third arginine to cysteine (Fig. 2C). Thus, we hypothesized that a loss of function of *argF* likely underlines the lack of immunity suppression by 10E10.

10.1128/mbio.03424-22.2 FIG S2 A screen of P. aeruginosa PAO1 transposon insertion mutants in candidate genes with point mutations in *P. simiae* WCS417 10E10 identified *argF*, which cannot acidify seedling exudates. Genome sequencing of 10E10 identified 35 non-synonymous candidate genes (Data Set S1, Table S1). Orthologs were identified in PAO1. *argF*::Tn5-1/-2: PS417_05595, ornithine carbamoyltransferase; *maeB*: PS417_01950, malate dehydrogenase; PA4465: PS417_04330, glmZ(sRNA)-inactivating NTPase; PA0214: PS417_26590, malonate decarboxylase subunit epsilon; PA0575: PS417_25885, diguanylate cyclase; and PA0148: PS417_03205, adenine deaminase. Statistics were calculated by using two-way ANOVA and Tukey’s HSD. Error bars represent mean +/− SD, and * indicate differences at *P* < 0.05. Download FIG S2, PDF file, 0.2 MB. Copyright © 2023 Liu et al. 2023 Liu et al. https://creativecommons.org/licenses/by/4.0/ This content is distributed under the terms of the Creative Commons Attribution 4.0 International license.

We tested whether a loss of *P. simiae* WCS417 *argF* could explain the inability of the 10E10 mutant to suppress immunity. First, we confirmed the C to T mutation in the *argF* catalytic site in the 10E10 mutant by PCR. We then complemented the 10E10 mutant with *argF* expressed by its native promoter in the pBBR1MCS-5 plasmid, and found that expression of *argF* under its native promoter (*argF_pro_*-*argF*) rescued the 10E10 mutant, and the complemented strain suppressed flg22-triggered *CYP71A12_pro_:GUS* expression, resulting in acidification of seedling exudates to a similar degree as wildtype WCS417 (Fig. 2A and B). We made a clean deletion of *argF* in WCS417, and found that the Δ*argF* mutant phenocopied the inability of the 10E10 mutant to suppress flg22-triggered *CYP71A12_pro_:GUS* expression, and could not acidify seedling exudates (Fig. 2A and B). These results illustrate that a loss of function of *argF* underlies the inability to suppress immunity by the 10E10 mutant.

### *pqqF* and *argF* pathways synergistically regulate rhizosphere pH.

**FIG 3:**
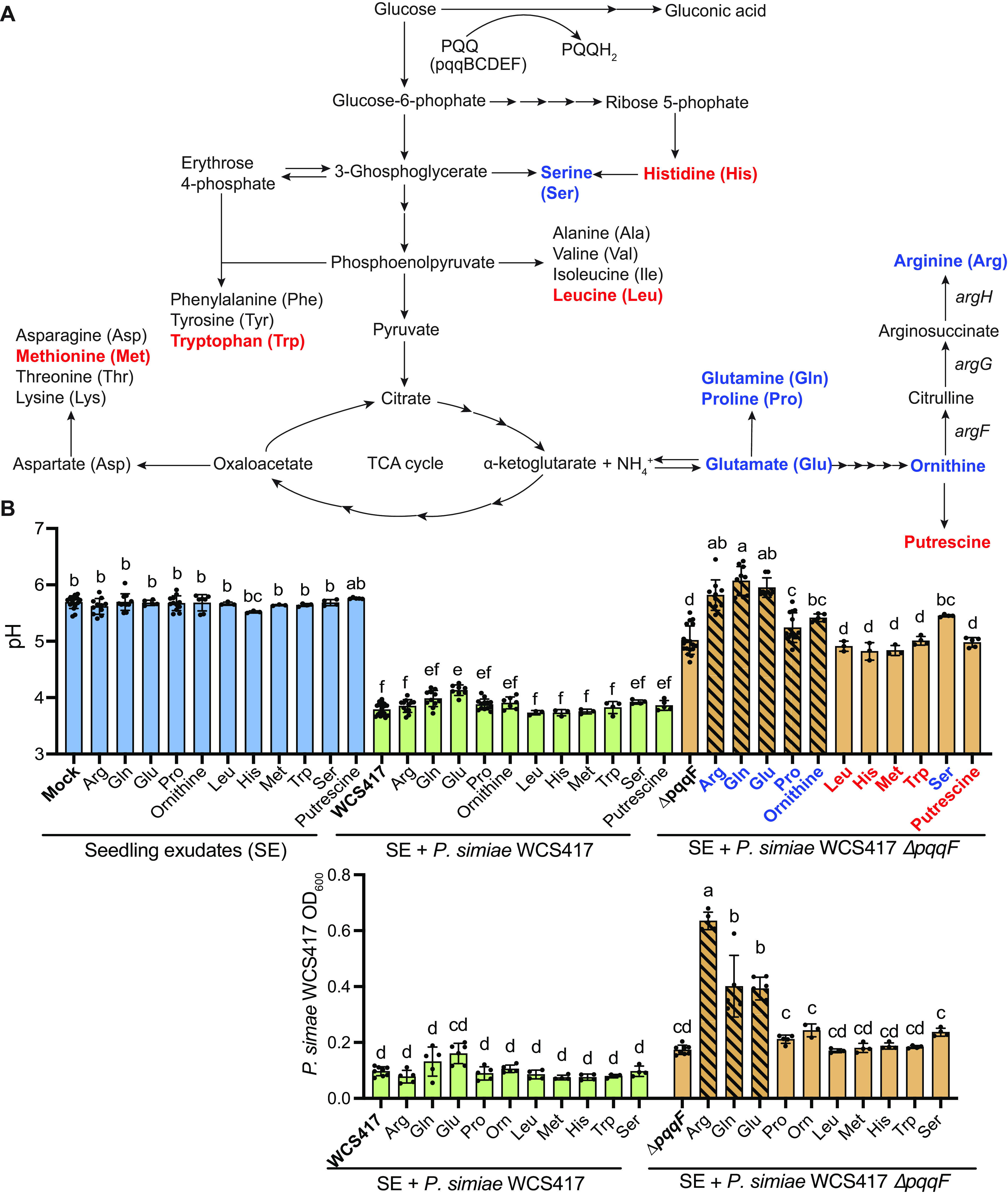
Accumulation of intermediates in the glutamate biosynthesis pathway contributes to rhizosphere alkalization. (A) Glutamate and gluconic acid biosynthesis pathways in Pseudomonas. (B) Inhibiting glutamate biosynthesis by providing arginine, proline, glutamine, and glutamate (striped bars) significantly raised the pH of WCS417 growing in seedling exudates. (C) Inhibiting glutamate biosynthesis by addition of exogenous arginine, glutamine, and glutamate resulted in overgrowth of the WCS417 *ΔpqqF* mutant. All the experiments were independently repeated at least 3 times. Statistics were calculated by using one-way ANOVA and Tukey’s HSD. Error bars represent mean ± SD, and letters indicate differences at *P* < 0.05.

Fungal pathogens can produce glutamate or glutamine to increase the local concentration of ammonia, and raise host pH to promote their own virulence (15, 16). As a result, it is an intriguing possibility that a loss of function mutation in *argF* may result in accumulation of alkaline arginine precursors such as ornithine, polyamines, and ammonia (Fig. 3A). A second, potentially confounding possibility is that arginine is limiting for growth in the rhizosphere, and so the *ΔargF* mutant may not be able to acidify because a lack of arginine is insufficient to support growth. If the former is true, then conditions resulting in accumulation of arginine precursors, such as addition of exogenous arginine, should result in accumulation of alkaline arginine precursors and rhizosphere alkalization. If the latter is true, and arginine is only required for growth, then addition of arginine should always result in increased growth and acidification.

We tested whether exogenous arginine would result in an increase or decrease in rhizosphere pH in *P. simiae* WCS417, the *ΔargF* mutant, and the *ΔpqqF* mutant. As exogenous arginine should suppress *argF* expression, it should mimic the *argF* mutant, and result in accumulation of alkaline precursors. We found that there was no effect on pH following the addition of 1 mM exogenous arginine in wildtype *P. simiae* WCS417 growing in seedling exudates (Fig. 2B). However, exogenous arginine fully restored acidification by both the 10E10 and Δ*argF* mutants, indicating that their inability to acidify is at least partly due to limited availability of arginine (Fig. 2B). Indeed, we confirmed that both mutants have growth defects in seedling exudates, which can be rescued by addition of exogenous arginine (Fig. 2D and E). Intriguingly, we found that arginine resulted in a significant increase in the pH of a *ΔpqqF* mutant growing in seedling exudates (Fig. 2B). This suggests that inhibition of *argF* may indeed result in increased pH through accumulation of arginine precursors, but that this effect may be masked by the large amount of gluconic acid produced by *P. simiae* WCS417.

*P. simiae* WCS417 produces large amounts of gluconic acid (8), and we only observed an increase in pH with application of exogenous amino acids in the *ΔpqqF* mutant (Fig. 2B). As a result, we hypothesized that *argF* might be necessary for lowering pH, but that gluconic acid might mask the effect of *argF*. If this is the case, we would expect that exogenous arginine would restore growth, but not lower the pH of a double *ΔpqqFΔargF* mutant growing in seedling exudates. In contrast, if *argF* only contributes to growth, addition of exogenous arginine should restore pH of the *ΔpqqFΔargF* mutant to the level of the single Δ*pqqF* mutant. Consistent with a role of ArgF in rhizosphere acidification, we found that addition of exogenous arginine fully restored growth of a *ΔpqqFΔargF* or *ΔargF* mutants to wildtype levels (Fig. 2E). However, arginine addition to the *ΔpqqFΔargF* double mutant resulted in significantly higher rhizosphere pH than the *ΔpqqF* mutant alone (Fig. 2B). These data indicate that *argF* is required for regulation of pH in the rhizosphere independent of growth.

We initially observed that the *P. simiae* WCS417 *ΔpqqF* mutant could not acidify the rhizosphere to a level that should result in full suppression of *CYP71A12_pro_:GUS* expression. However, the mutant can still fully suppress *CYP71A12_pro_:GUS* expression, suggesting a pH-independent mechanism of immunity suppression. While exogenous arginine resulted in a rhizosphere pH of 5.8, at which we would predict no suppression of the *CYP71A12_pro_:GUS* reporter (Fig. 1C), we found that arginine treatment of the *ΔpqqFΔargF* double mutant fully restored immunity suppression (Fig. S3). These data indicate that, while low pH is sufficient to suppress immunity, it is not necessary in WCS417, again supporting that there is a second, pH-independent mechanism of immunity suppression by *P. simiae* WCS417.

10.1128/mbio.03424-22.3 FIG S3 Addition of arginine to the *P. simiae* WCS417 *ΔargF ΔpqqF* mutant restores immunity suppression, but not acidification. Exogenous arginine had no effect on the ability of wild type *P. simiae* WCS417 to suppress expression of the *CYP71A12_pro_:GUS* reporter in the presence of flg22, but restored the immunity suppression by the 10E10 mutant, the *ΔargF* mutant, and the *ΔpqqFΔargF* double mutant. The addition of arginine to the Δ*pqqF* and *pqqFΔargF* double mutant fails to restore acidification (Fig. 2B). Download FIG S3, JPG file, 0.3 MB. Copyright © 2023 Liu et al. 2023 Liu et al. https://creativecommons.org/licenses/by/4.0/ This content is distributed under the terms of the Creative Commons Attribution 4.0 International license.

### Ornithine accumulation contributes to rhizosphere alkalization.

To test the hypothesis that accumulation of alkaline precursors of arginine, such as glutamate and ammonia could result in an increase in rhizosphere pH (Fig. 3A), we added arginine, proline, or glutamine to seedling exudates containing bacteria, which should result in an accumulation of their precursors, glutamate, and ammonia (17). We also tested the addition of exogenous methionine, leucine, tryptophan, serine, and histidine (which should not affect glutamate catabolism), as controls. To avoid confounding our findings with acidification through gluconic acid biosynthesis, we added amino acids to the *P. simiae* WCS417 *ΔpqqF* mutant. We found that exogenous arginine, proline, glutamine, serine and glutamate, but not methionine, leucine, tryptophan, or histidine, significantly raised the pH of the *ΔpqqF* mutant close to the level of mock seedling exudates (Fig. 3B). Moreover, we found this alkalization phenotype is even more dramatic in PAO1 as arginine, glutamine, and glutamate raised the pH of seedling exudates inoculated with PAO1 *pqqF*::Tn5 to around 8.0 (Fig. S4). These data indicate that, similar to pathogenic fungi, exogenous arginine, proline, glutamine, or glutamate likely result in rhizosphere alkalization through ammonia accumulation.

10.1128/mbio.03424-22.4 FIG S4 Addition of amino acids downstream of glutamine biosynthesis raised the pH of P. aeruginosa PAO1 growing in seedling exudates. Seedling exudates were treated with 1 mM amino acids and mock treated, or treated with PAO1 or the *pqqF::*Tn5 mutant. This showed that arginine, proline, glutamine, and glutamate significantly raised the pH of the *pqqF* mutant growing in seedling exudates. All the experiments were independently repeated at least 3 times. Statistics were calculated by using one-way ANOVA and Tukey’s HSD. Error bars represent mean +/− SD, and letters indicate differences at *P* < 0.05. Download FIG S4, PDF file, 0.4 MB. Copyright © 2023 Liu et al. 2023 Liu et al. https://creativecommons.org/licenses/by/4.0/ This content is distributed under the terms of the Creative Commons Attribution 4.0 International license.

To genetically test whether a loss of arginine, proline, glutamine, or glutamate could specifically result in increased pH of seedling exudates, we selected 6 mutants that have insertions in the genes that are required for amino acid biosynthesis from the P. aeruginosa PAO1 transposon insertion library (Data Set S1, Table S3). We found that *proA*::Tn5 (deficient in proline biosynthesis) and *gltB*::Tn5 (deficient in glutamine biosynthesis) neither acidify seedling exudates, nor suppress PTI (Fig. S5), which is consistent with the glutamate biosynthetic pathway being required for rhizosphere acidification. Two additional amino acid insertions in *metZ*::Tn5 (deficient in methionine biosynthesis) and *leuC::Tn5* (deficient in leucine biosynthesis) also resulted in a loss of acidification of seedling exudates and immunity suppression of seedlings, suggesting that leucine and methionine may be limiting for bacterial growth in the rhizosphere (Fig. S5). In addition, *serA*:: Tn5 (deficient in serine biosynthesis) suppressed host immunity and had decreased pH of seedling exudates, indicating that the rhizosphere may contain enough serine to support bacterial growth (Fig. S5). Interestingly, a *hisB*::Tn5 mutant (deficient in histidine biosynthesis) can also acidify seedling exudates but induced immunity on its own, further indicating that low pH alone is not sufficient for immunity suppression (Fig. S5). Collectively, these data confirm that the rhizosphere is deficient in glutamate and downstream amino acids, and so bacteria must actively synthesize these in the rhizosphere.

10.1128/mbio.03424-22.5 FIG S5 Bacterial auxotrophs cannot suppress plant immunity or acidify the rhizosphere. (A) P. aeruginosa PAO1 amino acid auxotrophic mutants fail to acidify the seedling exudates with the exception of *hisB*::Tn5 and *serA*::Tn5 in PAO1. (B) Amino acid auxotrophic mutants fail to suppress flg22-induced *CYP71A12_pro_:GUS* expression, with the exception of *serA*::Tn5. The *hisB*::Tn5 PAO1 mutant induces *CYP71A12_pro_:GUS* expression in the absence of exogenous flg22 treatment. All the experiments were independently repeated 3 times. Statistics were calculated by using one-way ANOVA. Error bar represents mean +/− SD. Download FIG S5, JPG file, 0.4 MB. Copyright © 2023 Liu et al. 2023 Liu et al. https://creativecommons.org/licenses/by/4.0/ This content is distributed under the terms of the Creative Commons Attribution 4.0 International license.

As fungal alkalization through glutamate secretion is accompanied by increased fungal growth and virulence (18), we tested whether an increase in pH might result in bacterial overgrowth. We observed that arginine, glutamine, and glutamate-mediated alkalization were accompanied by dramatic bacteria overgrowth of both the WCS417 and PAO1 Δ*pqqF* mutants (Fig. 3C and Fig. S6). To determine if immunity was suppressed at high pH, we tested whether flg22 could still trigger *CYP71A12_pro_:GUS* expression at pH 8, and found that expression still occurred (Fig. S7), indicating that overgrowth is occurring at a pH that is not sufficient to suppress immunity. These data indicate that pH correlates with bacteria growth, and that by limiting certain amino acids, it is possible that plants can control the rhizosphere pH and bacterial growth via arginine biosynthesis.

10.1128/mbio.03424-22.6 FIG S6 The addition of arginine, glutamine, or glutamate resulted in overgrowth of the P. aeruginosa *pqqF* mutant. P. aeruginosa *PAO1* or the *pqqF:*Tn5 mutant were grown in seedling exudates, and inoculated with 1 mM of the indicated amino acid. Statistics were calculated by using one-way ANOVA and Tukey’s HSD. Error bars represent mean +/− SD, and letters indicate differences at *P* < 0.05. Download FIG S6, PDF file, 0.4 MB. Copyright © 2023 Liu et al. 2023 Liu et al. https://creativecommons.org/licenses/by/4.0/ This content is distributed under the terms of the Creative Commons Attribution 4.0 International license.

10.1128/mbio.03424-22.7 FIG S7 Flg22-triggered immunity is intact at pH 8. We found, upon raising the pH to 8.0 (the pH associated with bacterial overgrowth) with KOH, that flg22-triggered expression of the *CYP71A12_pro_:GUS* promoter, meaning it is not suppressed. Download FIG S7, PDF file, 0.9 MB. Copyright © 2023 Liu et al. 2023 Liu et al. https://creativecommons.org/licenses/by/4.0/ This content is distributed under the terms of the Creative Commons Attribution 4.0 International license.

To test our prediction that rhizosphere alkalization is due to accumulation of ammonia from inhibiting the arginine biosynthesis pathway, we measured the ammonium concentration in seedling exudates. In contrast to our prediction that addition of arginine, glutamine, or glutamate would increase ammonium, we found that the *ΔpqqF* mutant consumes more ammonium than wild type bacteria in the presence of these amino acids (Fig. S8). This suggests that, when arginine biosynthesis is inhibited, ammonia is converted to a distinct compound that contributes to rhizosphere alkalization.

10.1128/mbio.03424-22.8 FIG S8 Ammonium concentration does explain alkalinization in the *P. simiae* WCS417 *ΔpqqF* mutant growing in seedling exudates treated with arginine. Ammonium was quantified in seedling exudates (mock), or seedling exudates grown with *P. simiae* WCS417 or the Δ*pqqF* mutant. Seedling exudates were treated with 1 mM of the indicated amino acid. Statistics were calculated by using one-way ANOVA and Tukey’s HSD. Error bars represent mean +/− SD, * indicate differences at *P* < 0.05, and *** indicate differences at *P* < 0.01. Download FIG S8, PDF file, 0.4 MB. Copyright © 2023 Liu et al. 2023 Liu et al. https://creativecommons.org/licenses/by/4.0/ This content is distributed under the terms of the Creative Commons Attribution 4.0 International license.

**FIG 4:**
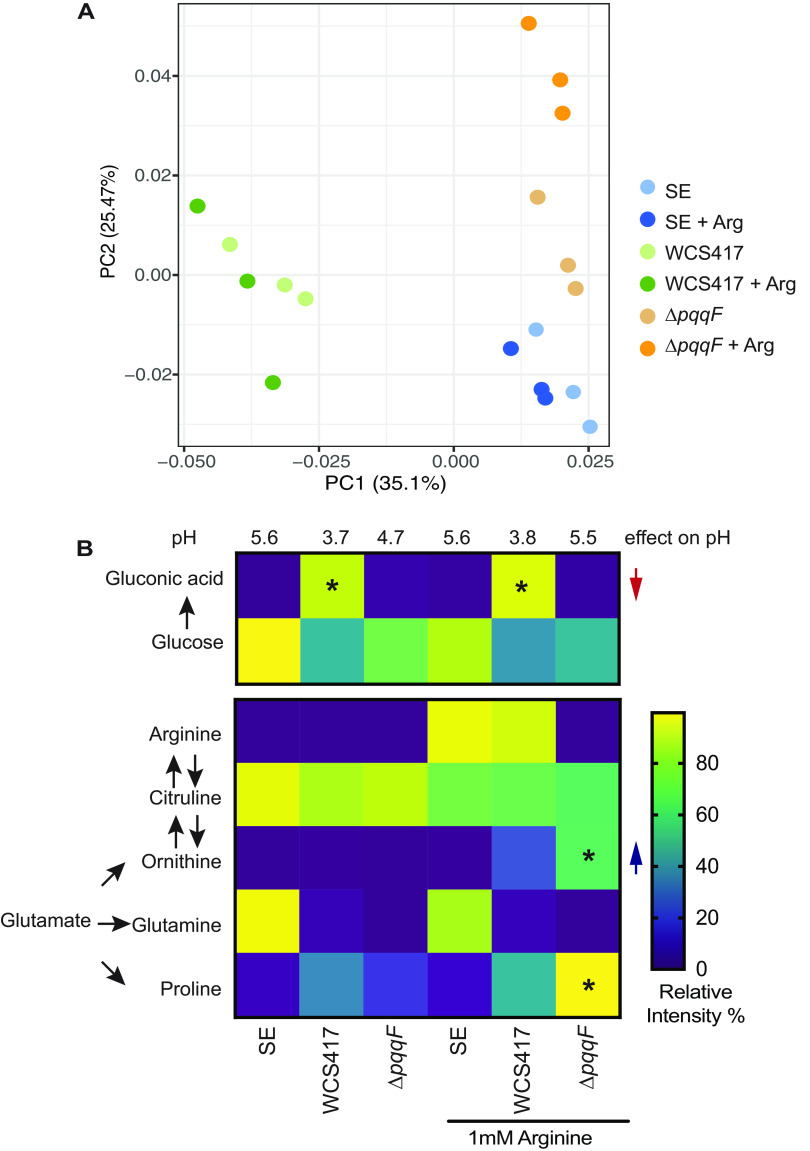
Untargeted metabolomics identified ornithine accumulation is associated with rhizosphere alkalization. (A) Untargeted metabolomics revealed that genotype has the largest impact on the metabolite profiles as seedling exudates, or seedling exudates treated with *P. simiae* WCS417 or the *P. simiae* WCS417. The *ΔpqqF* mutant showed distinct metabolite profile, regardless of addition of arginine. PCoA calculated by Bray-Curtis distances of the pooled samples that appeared in both HILIC positive mode and negative mode. Statistical significance was calculated by permutational multivariate ANOVA (*n* = 3 for each treatment). (B) Ornithine accumulates in the presence of arginine uniquely in the *ΔpqqF* mutant, indicating that *ΔpqqF* mutant converted into exogenous arginine to ornithine, an alkaline molecule. All the experiments were independently repeated at least 3 times. Statistics were calculated by using one-way ANOVA and Tukey’s HSD. Error bars represent mean ± SD, * indicate differences at *P* < 0.05, and *** indicate differences at *P* < 0.01.

To uncover the compound that caused the rhizosphere alkalization, we performed untargeted metabolomics of seedling exudates, or seedling exudates containing *P. simiae* WCS417 or the *ΔpqqF* mutant with or without arginine. We found that mock and bacterial treatments were clearly separated in the pooled principal coordinates analysis (PCoA), as shown in PC1 (*P* < 0.001) (Fig. 4A). We found that the wild type WCS417 and the *ΔpqqF* mutant also have distinct metabolite profiles, regardless of the presence of exogenous arginine, as shown in PC2 (*P* < 0.002), indicating that loss of *pqqF* has a significant impact on the bacterial metabolism (Fig. 4A). While arginine did not cause a significant global change across all conditions (*P < *0.1), we saw that arginine affected the metabolite profile of the *ΔpqqF* mutant to a greater degree than it did when added to wildtype *P. simiae* WCS417, as shown in PC2 (Fig. 4B).

When we observed individual metabolites, we found that, while wildtype bacteria produced a large amount of gluconic acid, the *ΔpqqF* mutant produced no detectable gluconic acid (Fig. 4B). We also found that, while seedling exudates with wild type WCS417 contain similar amount of arginine as seedling exudates with no bacteria, the *ΔpqqF* mutant completely depleted the exogenous arginine, indicating that the *ΔpqqF* mutant converted the exogenous arginine to a distinct compound (Fig. 4B). Although we did not observe significant differences of citrulline between the WCS417 and *ΔpqqF* mutant, addition of exogenous arginine resulted in a reduction in the overall amount of citrulline, indicating feedback inhibition of arginine on *argF* (13) (Fig. 4B).

We queried our untargeted metabolomics data for compounds that uniquely accumulated in the *P. simiae* WCS417 *ΔpqqF* mutant in the presence of arginine (Data Set S1). We found that the *ΔpqqF* mutant uniquely accumulates significantly higher ornithine and proline than the wild type in the presence of exogenous arginine (Fig. 4B). While proline has a neutral pKa, ornithine is an alkaline non-proteogenic amino acid with pKa of 10.29, and it is a substrate of ArgF in the arginine biosynthesis pathway. Although spermidine and putrescine are also alkaline polyamine compounds derived from ornithine, we only detected trace amount of putrescine, spermidine, and spermine that were too low to be quantified. Additionally, exogenous arginine did not significantly change the amount of malic acid in the *ΔpqqF* mutant relative to wildtype WCS417 (Data Set S1), suggesting that addition of exogenous arginine unlikely affected the TCA cycle. These data suggest that, upon arginine addition to the *ΔpqqF* mutant, the accumulation of ornithine underlies the rhizosphere alkalization.

To test the possibility that ornithine, but not polyamines, resulted in increase in rhizosphere pH, we added exogenous polyamines or ornithine to WCS417 or the *ΔpqqF* mutant. We found that the addition of exogenous ornithine or putrescine had no effect on the pH of WCS417 in seedling exudates, and that ornithine, but not putrescine, resulted in a significant increase in the pH of the *ΔpqqF* mutant (Fig. 3B). However, we observed no effect of ornithine addition to the wildtype P. aeruginosa PAO1 or the PAO1 *ΔpqqF* mutant (Fig. S4), indicating that the compound that results in alkalization might differ in PAO1 from WCS417.

### Acidification-mediated immunity suppression is peptide-specific in roots.

Our data show that Pseudomonas have multiple, partially redundant mechanisms to acidify the rhizosphere, indicating that maintenance of correct pH may be critical to establishing plant immune homeostasis. Previously, we found that acidification to pH 3.7 partially blocked flg22-mediated induction of the *MYB51_pro_:GUS* reporter gene, but had no effect on the expression of SA-triggered *NPR1_pro_:GUS* and *MYB72_pro_:GUS* triggered by WCS417 or WCS358 at pH 3.7 in roots, indicating that acidification can only impair a section of plant innate immunity (8). Thus, we wondered whether acidification suppression of immunity affects MAMP binding to receptors, or affects downstream signaling.

**FIG 5:**
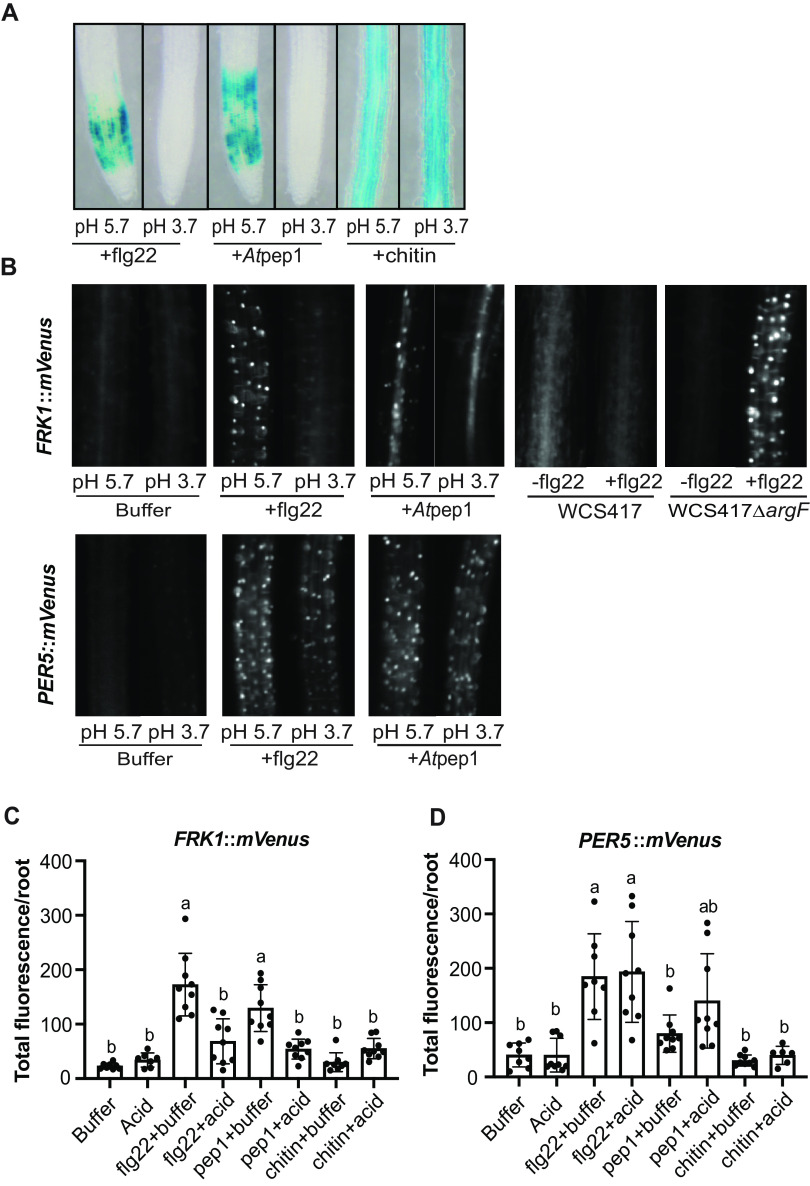
Acidification only suppresses a sector of plant innate immunity. (A) Acidification blocks flg22- but not Atpep1- or chitin-triggered *CYP71A12_pro_:GUS* expression. (B) Acidification and WCS417, but not the WCS417 *ΔargF* mutant can block flg22-induced *FRK1::mVenus* fluorescent reporter expression. (C-D) Acidification blocks flg22-induced *FRK1::mVenus* expression, but not *PER5::mVenus* expression. All the experiments were independently repeated 3 times. Statistics were calculated by using one-way ANOVA and Tukey’s HSD. Error bars represent mean ± SD, and letters indicate differences at *P* < 0.05.

If acidification specifically blocks flg22-induced immunity, then low pH should not affect other MAMP-triggered immunity pathways. We tested the effect of acidification on the damage-associated molecular pattern *At*pep1, which is BAK1-dependent (19, 20), and chitin, a sugar polymer from fungal cell walls, which is BAK1-independent in *Arabidopsis* (21, 22). All 3 MAMPs (flg22, *At*pep1, and chitin) share a MAPK cascade (23–25). We tested whether acidification can also suppress chitin- and *At*pep1-triggered immunity using the MAMPs marker gene reporters *CYP71A12_pro_:GUS*. We found that pH 3.7 abolished *At*pep1-, but not chitin-triggered, expression of the *CYP71A12_pro_:GUS* reporter (Fig. 5A). These data indicate that acidification likely interferes with the peptide-triggered immunity.

To test if low pH affects specific defense responses, we tested whether expression of multiple PTI-induced genes was blocked by using additional PTI-inducible reporters. We used *PER5* (AT1G14550), which is a peroxidase (26), and *FRK1* (AT2G19190), which encodes a LRR receptor kinase (27). Both genes are flg22 and *At*pep1-inducible in the root (28–30). We found that both flg22- and *At*pep1-induced *FRK1*::mVenus expression, which was significantly suppressed by low pH (Fig. 5B, C). In contrast, lower pH did not affect flg22-induced *PER5*::*mVenus* expression or *At*pep1-induced *PER5*::*mVenus* expression (Fig. 5D). These results suggest that acidification can only affect a sector of PTI, and rhizosphere pH may be critical to determine the baseline setting on the plant immune thermostat.

## DISCUSSION

Here we report a forward genetic screen that identified a bacterial gene ornithine carbamoyltransferase *argF* from P. simiae WCS417 that is required for host immunity suppression, colonization, and acidification. The Δ*argF* mutant is auxotrophic, and exogenous arginine restored Δ*argF*-mediated host immunity suppression, colonization, and acidification to wildtype levels. This indicates that amino acid biosynthesis plays an important role in rhizosphere colonization. This is not the first time that amino acid biosynthesis has been shown to be necessary for root colonization (7, 31). Interestingly, a previous TnSeq screen found that amino acid auxotrophs, including insertions in *argF* in *P. simiae* WCS417, exhibited enhanced fitness in the *Arabidopsis* rhizosphere (32). We suspect the difference between these findings may be because of a community of transposon insertion mutants in a TnSeq screen, where the presence of other mutants could potentially provide amino acids in *trans* to auxotrophs. In fact, metabolic exchange, including amino acid cross-feeding among microbes, is characteristic and reciprocal in a microbial community (33–35). Our data suggest that the rhizosphere may be limiting in many amino acids, and that by synthesizing certain amino acids, bacteria will alter the rhizosphere pH and affect plant immune homeostasis.

To disentangle the role of amino acid biosynthesis in rhizosphere acidification from their role in growth, we supplied different amino acids to Pseudomonas *pqqF* mutants, which cannot produce gluconic acid but retain some rhizosphere acidification (Fig. 2B). We found that only arginine, proline, ornithine, glutamine, and glutamate caused rhizosphere alkalization, and arginine, glutamine, and glutamate caused bacterial overgrowth in the *pqqF*-deficient mutants. Metabolic profiling suggests that alkalization by Pseudomonas was likely the consequence of ornithine accumulation, an alkaline non-proteogenic amino acid, and the substrate of ArgF. Interestingly, alkalization is associated with overgrowth of *pqqF*-deficient mutants, which is reminiscent of alkalization-mediated invasive growth in fungi (18). However, Pseudomonas does not utilize ammonia for alkalization, as ammonia-driven alkalization requires carbon deprivation (15, 16). We found alkalization in the presence of glucose, as the *ΔpqqF* mutant retains more glucose than the wild type because it does not convert glucose to gluconic acid. We found that the overgrowth of the *ΔpqqF* mutant in the presence of arginine was accompanied by significantly more glucose consumption than the *ΔpqqF* mutant without exogenous arginine. These data suggest that maintaining a balance of carbon and nitrogen containing compounds is essential for pH homeostasis and bacterial growth regulation.

We found that, while *P. simiae* WCS417 produces gluconic acid and significantly acidifies the rhizosphere, it is not necessary for immunity suppression in *P. simiae* WCS417, indicating there is a distinct mechanism of immunity suppression. The *ΔpqqF* mutant and the *ΔpqqFΔargF* mutant supplemented with exogenous arginine caused an increased in rhizosphere pH, but still suppressed immunity. This indicates that there are additional pH-independent mechanisms for host immunity manipulation in *P. simiae* WCS417.

Rhizosphere acidification seems to be a general characteristic of many root-associated microbes (8, 9). Thus, rhizosphere acidification could be a conserved evolutionary trait of root-associated microbes. However, suppression of host immunity may also open the window for pathogens. In this study, we found that acidification can only dampen a sector of immunity, indicating that plant must also evolve novel mechanisms to counteract acidification-mediated immunity suppression, which may act as a selection force for microbial colonization. Recently, roots were shown to contain both acidic (early elongation zone and root tip) and alkaline domains (late elongation zone/root hair zone) (36). Interestingly, we found that acidic pH does not suppress chitin-triggered immunity in the maturation zone, suggesting that acidification-mediated immunity suppression might be zone-dependent (Fig. 5) rather than ligand dependent.

**FIG 6:**
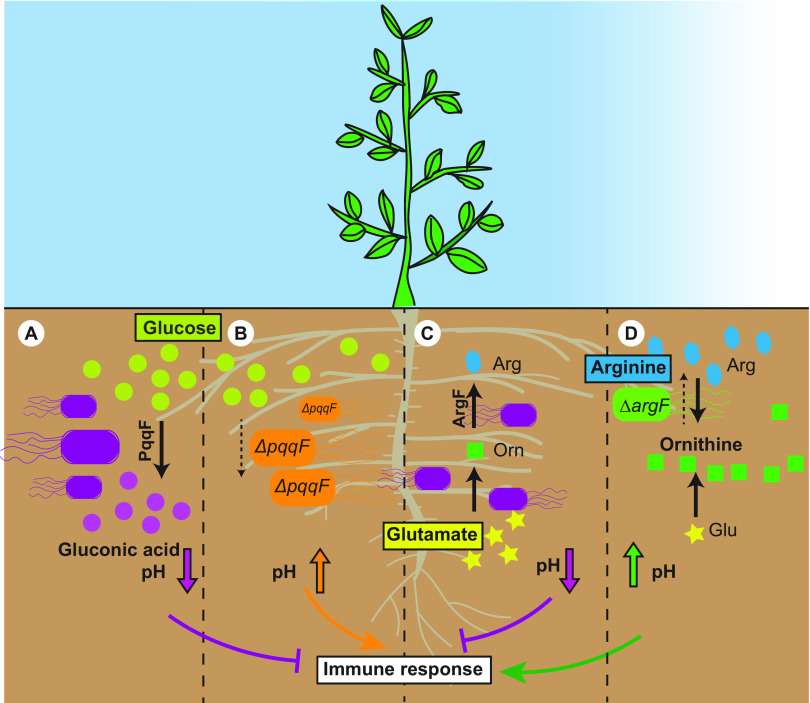
Model of bacteria-mediated rhizosphere acidification and acidification-mediated plant innate immunity suppression. (A) Bacteria convert plant-derived glucose to gluconic acid in order to acidify the rhizosphere and suppress plant innate immunity. (B) Loss of *pqqF* in bacteria (orange bacteria) results in a loss of gluconic acid biosynthesis, loss in acidification, and, in some cases, the inability to suppress plant immunity. (C) Bacteria actively synthesize certain amino acids (i.e., arginine) to avoid buildup of alkaline precursors. (D) When plants provide sufficient arginine, ornithine builds up, resulting in rhizosphere alkalization and significant bacterial overgrowth. Overall, this work suggests that maintaining an amino acid-glucose balance is crucial for regulating plant rhizosphere microbiome assembly.

Collectively, host immunity suppression is crucial for host colonization of both commensals and pathogens. Crosstalk between the microbes and the plant immune system is an ongoing process. Our results highlight that, apart from serving as colonization factors and nutrients, bacterial amino acid biosynthesis plays a novel dual role in the rhizosphere acidification and host immunity suppression (Fig. 6). As acidification quenches only a sector of host immunity, it is clear that more mechanisms of host immunity suppression are still to be discovered.

## MATERIALS AND METHODS

### Plant materials and growth conditions.

We used Arabidopsis thaliana wild type Col-0, *CYP71A12_pro_:GUS* reporter (10) and *FRK1*::*mVenus*, *PER5*::*mVenus* (29) in our study. All the plant material used in our study were grown in the same condition, which was in a climate-controlled growth room at 21^°^C, 16 h light/8 h dark cycle with light intensity of 100 μM. Plants were grown in the ½ X Murashige and Skoog (MS) media with 1% MES [2-(N-morpholino) ethanesulfonic acid] with 0.5% sucrose, adjusted by KOH to a pH of 5.7 (10).

### Bacterial strains and growth condition.

Strains that were used in this study are listed in Data Set S1, Table S2. P. simiae WCS417 and mutants were cultured overnight in LB or King’s B at 28°C with shaking at 180 rpm. P. aeruginosa PAO1 was cultured overnight in LB at 37°C with shaking at 180 rpm. PAO1 transposon insertion mutants were obtained from the 2-allele PAO1 transposon insertion library (12). Wildtype PAO1 and the transposon insertion mutants used in this study were cultured in LB with 25 μg/mL tetracycline at 37°C. Escherichia coli were cultured in 37°C with 15 μg/mL or 100 μg/mL gentamicin, depending on the experiment.

### ß-glucosidase histochemical assays.

The reporter lines *CYP71A12_pro_:GUS* in the *Arabidopsis* Col-0 genetic background contains the *CYP71A12* promoter driving the expression of the ß-glucosidase (GUS) reporter gene (10). Seeds were grown in 48-well plates for 1 week after surface sterilization, and were grown in the condition described above. Each well contained 300 μL ½x MS media and 0.5% sucrose. On day 8, the media was replaced by fresh ½x MS with 0.5% sucrose media. Bacteria were grown overnight in LB, washed in 10 mM MgSO_4_, and serially diluted to an OD_600_ of 0.02 in 10 mM MgSO_4_. On day 9, 30 μL bacteria were added to each well (final OD_600_ of 0.002), and the plates were returned to the growth room for at least 18 h before adding flg22. On day 10, 500 μM flg22 was added to a final concentration of 500 nm, and the media was replaced with GUS staining solution after 4.5 h of incubation. The GUS staining solution was made fresh at a final concentration of 0.5 M sodium phosphate buffer (pH 7), 0.5M EDTA, 50 mM potassium ferricyanide, 50 mM potassium ferrocyanide, 50 mM X-Gluc (5-bromo-4-chloro-3-indolyl-beta-d-glucuronic acid), and 10 μL Triton X-100. Plates were then incubated at 37°C without light until the control roots treated with flg22 developed a visible blue color (approximately 3 to 4 h). Finally, to clear the tissue, the GUS stain was replaced with 95% ethanol, and washed with water afterwards. Images were taken with a Macro Zoom Fluorescence Microscope MVX10.

### Bacterial growth curves.

Overnight cultures of bacteria were grown in LB, then were serially diluted to an OD_600_ of 0.2 in 10 mM MgSO_4_ for growth curves. Growth curves were performed by adding 10 μL of the diluted culture to 90 μL rich media (LB), minimal media (M9 salts supplemented with 30 mM succinate), or seedling exudates (M9 salts supplemented with 30 mM succinate, with or without 1 mM arginine). Bacteria growth was quantified by measuring OD_600_ on a Versamax plate reader (Molecular Devices). Data presented in this study represent the average of 3 biological replicates.

### WCS417 EMS mutant library construction and screening.

P. simiae WCS417r (rif resistant variant) was mutagenized by spinning down and washing an overnight culture, and exposing it to 1, 2, or 4% EMS for 1 h. Mutagenized cells were plated on King’s B with 50 μg/mL streptomycin, and it was found that after treatment with 4% EMS there was ~ 100-fold increase in the number of resistant cells relative to the parental strain, so these cells were used for library construction. For library construction, mutagenized cells were plated on LB + rifampicin 50 μg/mL, and individual colonies were placed into wells of 96-well deep well plates in LB media. Each plate contained 92 EMS mutants, and 4 wells contained the parental strain as positive controls. After overnight growth, 75 μL of LB containing library bacteria were pipetted into a fresh 96-well plate, and 25 μL of 80% glycerol was added. The library was stored at −80°C.

To screen the library, seedlings were grown in 96-well plates in MS media as described above. Three seedlings were grown in each well containing 100 μL MS media. The library was stamped onto rectangular plates containing solid LB media, and then subcultured into 96-well deep bottom plates containing LB. After overnight growth, the OD_600_ of 4 independent wells was measured, and the average was taken. This was used to calculate an approximate dilution factor to dilute all 96 wells to a final OD_600_ = 0.05. A total of 10 μL of the diluted culture was added to each well, containing 90 μL MS, for a final average bacterial concentration of 0.005. The screen was repeated in duplicate, and candidates that failed to suppress immunity in both replicates were retested.

### Mapping WCS417 EMS mutations.

To map WCS417 EMS mutations, we sequenced the genomes of the parental line used to make the library, as well as the genomes of each individual mutant. Genomic DNA was extracted from WCS417 and the 10E10 EMS mutant with the Puregene Core Kit (Qiagen). A PE150 short insert library was prepared and sequenced on an Illumina HiSeq 2500 (Novogene). After adapter trimming with Cutadapt (37), reads were aligned to the WCS417 genome using the Bowtie2 (38) aligner with default parameters. Variant calling was performed with BCFtools (39). Low quality SNPs with a quality score under 20 were filtered out, and SNPs found in the parent strain were discarded from consideration.

### Bacteria mutant complementation.

Complementation of the 10E10 mutant was performed by PCR amplifying the coding sequence and native promoter of *argF* in WCS417. The PCR product containing HindIII and BamHI restriction sites was ligated to the plasmid pBBR1MSC5, and the ligation product was transformed into the competent cell *E.coli* DH5α, and plated on gentamicin 100 μg/mL plates for selection of positive colonies. Positive colonies containing the *proargF*:pBBR1MSC5 construct were confirmed by colony PCR, and further confirmed by Sanger sequencing. The confirmed pBBR1MCS-5::Pro*_argF_*-*argF* construct was then transformed into the 10E10 mutant.

### Generation of deletion mutants in WCS417.

Clean deletions of *argF* or *pqqF* in WCS417 were made using a double-recombination method in Gram-negative bacteria using counter selection with *sacB* (40). Two sets of primers were designed to amplify the 500 bp flanking region upstream and downstream of *argF* or *pqqF*. Primer 1 with the restriction enzymes site HinIII, and primer 4 with the restriction enzyme site BamHI are the left and right primers of the upstream and downstream flanking region of the target gene, respectively. Primer 2 and primer 3 are the right and left primers of the upstream and downstream flanking region of the target gene, respectively. Both primer 2 and primer 3 consist of 15 bp of region upstream and 15 bp of region downstream of the target gene. Thus, primer pairs primer 1 and primer 2, and primer 3 and primer 4 were used to amplify the 500 bp regions upstream and downstream of the target gene, respectively. Overlap PCR was performed with the upstream and downstream PCR products, which were digested with HindIII and BamHI, and ligated to the pEXG2 suicide vector containing *sacB* (41), and then transformed into *E.coli* DH5α. The positive colonies were selected for plating on LB plates with gentamicin 15 μg/mL, and then confirmed by colony PCR. The deletion constructs for *argF* or *pqqF* were further confirmed by Sanger sequencing. The confirmed *argF* or *pqqF* deletion constructs were then transformed into the competent SM10λ cells, and were selected on LB plates with gentamicin 15 μg/mL. Conjugation of the SM10λ containing the deletion construct and WCS417 was performed, and the transconjugants were selected on plates containing nalidixic acid 15 μg/mL and gentamicin 100 μg/mL. Positive colonies were re-streaked, and cultured overnight in plain LB. Cell pellets were diluted to 10X and 100X, and were each plated onto 10% sucrose plates and gentamicin 100 μg/mL plates to select for the second recombination. The Δ*argFΔpqqF* double mutant was made by conjugating the *ΔpqqF* mutant with the SM10λ strain containing the *argF*-pPEXG2 deletion construct, and the selection was performed as described above.

### Seedling exudates.

To generate seedling exudates, A. thaliana Col-0 seeds were grown in half strength MS media containing 0.5% sucrose for 7 days, as described above. The seedling exudates were collected from all the wells, and immediately syringe filtered with a 0.22 μm filter and frozen at −20°C.

### Amino acid solutions.

A total of 100 mM (100X) stock solutions of l-arginine, L-proline, l-glutamine, l-glutamate, l-ornithine, l-leucine, l-methionine, l-histidine, L-tryptophan, and l-serine were made in water and filter sterilized using a 0.22 μm filter, before storing at 4°C.

### pH assay in seeding exudates.

Bacteria were grown in LB overnight and were serially diluted to OD_600_ 0.02 in 10 mM MgSO_4_. Bacteria were inoculated into 24-well plates containing seedling exudates to a final OD of 0.002, and the plates were incubated in a 28°C or 37°C incubator for 18 h. Final concentration of 1 mM amino acids (100X dilution of stocks) were added when indicated. Then, 1 mL of culture was directly taken from each well, and the OD was measured by a spectrophotometer. Each experiment included 3 technical replicates, and was independently repeated at least three times.

### Ammonium quantification.

Ammonium concentration in the pH assay was measured by an Ammonia assay kit (Sigma). The kit provides reagents that reacts with ammonia/ammonium ions, which produces fluorescence signals that are proportional to the ammonia concentration in the sample. A total of 1 mL of each sample was taken from the pH assay and centrifuged for 5 min at 14,000 rpm. Then, 10 μL of the supernatant was used for ammonia quantification following the manufacturer’s instructions. Plates were read by a Spectramax plate reader (λ_ex_ = 360/λ_em_ = 450 nm).

### Metabolomic profiling.

The samples described above in the “pH assays in seedling exudates” were used for untargeted metabolomics analysis. A 60 μL aliquot of each media sample was diluted with 60 μL of acetonitrile (ACN), and vortexed for 10 sec. The mixture was then centrifuged at 14000 rpm for 15 min at 4°C. The supernatant was transferred to glass inserts in 2 mL autosampler vials for liquid chromatography-mass spectrometry (LC-MS) analysis. The analysis was performed using a Bruker Impact II Ultra-High Resolution Qq-Time-of-Flight mass spectrometer coupled to an Agilent 1290 Infinity Liquid Chromatography system. For each sample, 2 μL was injected onto an EMD Millipore SeQuant ZIC-pHILIC column (200 Å, 5 μm, 2.1 × 150 mm) for hydrophilic interaction chromatography (HILIC) separation. For negative ionization mode, the mobile phases (MPs) were 10 mM ammonium acetate in 95/5 water/ACN at pH 9.8 (MP A), and 95/5 ACN/water (MP B). The MPs for positive ionization were the same, except MP A was pH 4.8. The same LC gradient was used for both ionization modes at a flow rate of 0.150 mL/min. The separation gradient, described in percentage of MP B, started at 95%, dropped to 5% over 20 min, and then increased to 95% over 1 min; the column was equilibrated for 14 min between injections. A pooled quality control sample was injected at 5 different volumes for metabolic signal correction, and high-quality feature selection (42). Sodium formate was injected for mass calibration. The mass spectrometer was operated in Auto MS/MS mode. The ionization capillary voltage was 3.6 kV for negative mode, and 4.5 kV for positive mode. The nebulizer gas was 1.6 bar. The dry gas was 7 L/min, and the dry temperature was 220°C. The mass range collected was from 70 to 1500 *m/z* at 8 Hz. The collision energy was 20 to 50 eV. The acquired data were calibrated and processed with MS-DIAL (ver. 4.80). The resulting metabolite intensity tables were exported for statistical analysis.

### Fluorescence reporter imaging and quantification.

*FRK1*::mVenus and *PER5*::mVenus seedlings were grown in 600 μL of ½x MS media with 0.5% sucrose and a pH of 5.7 in 24-well plates. On day 8, the media was replaced with 540 μL of fresh ½x MS with 0.5% sucrose at a pH 5.7. On day 9, MAMPs were added to a final concentration of 500 μM flg22, 100 nM *At*pep1, or 0.1 mg/mL chitin were added. For low pH conditions, ½x MS with 0.5% sucrose with pH 3.7 were added, along with the elicitors described above, and incubated for 4.5 h. Images were taken with a Macro Zoom Fluorescence Microscope MVX10 microscope.
