# Supplementary figures and images for: Amino Acid Availability Determines Plant Immune Homeostasis in the Rhizosphere Microbiome
Source: mBio. 2023 Feb 14;14(2):e03424-22. doi: 10.1128/mbio.03424-22 (PMC10127609; doi:10.1128/mbio.03424-22)

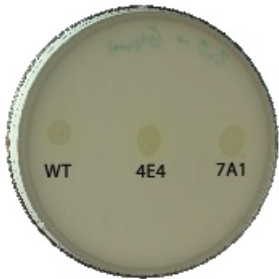

1.5% Glycerol

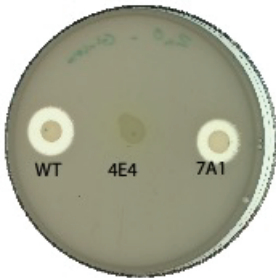

1% Glucose

Supplement: FIG S1 [file mbio.03424-22-s0001.pdf]

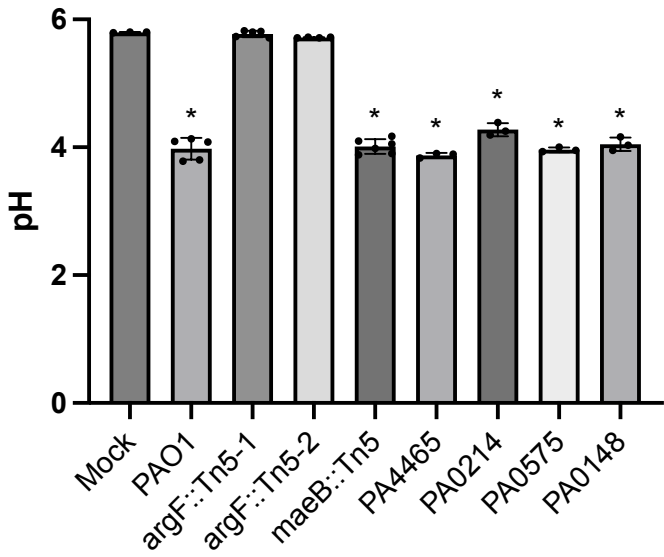

Supplement: FIG S2 [file mbio.03424-22-s0002.pdf]

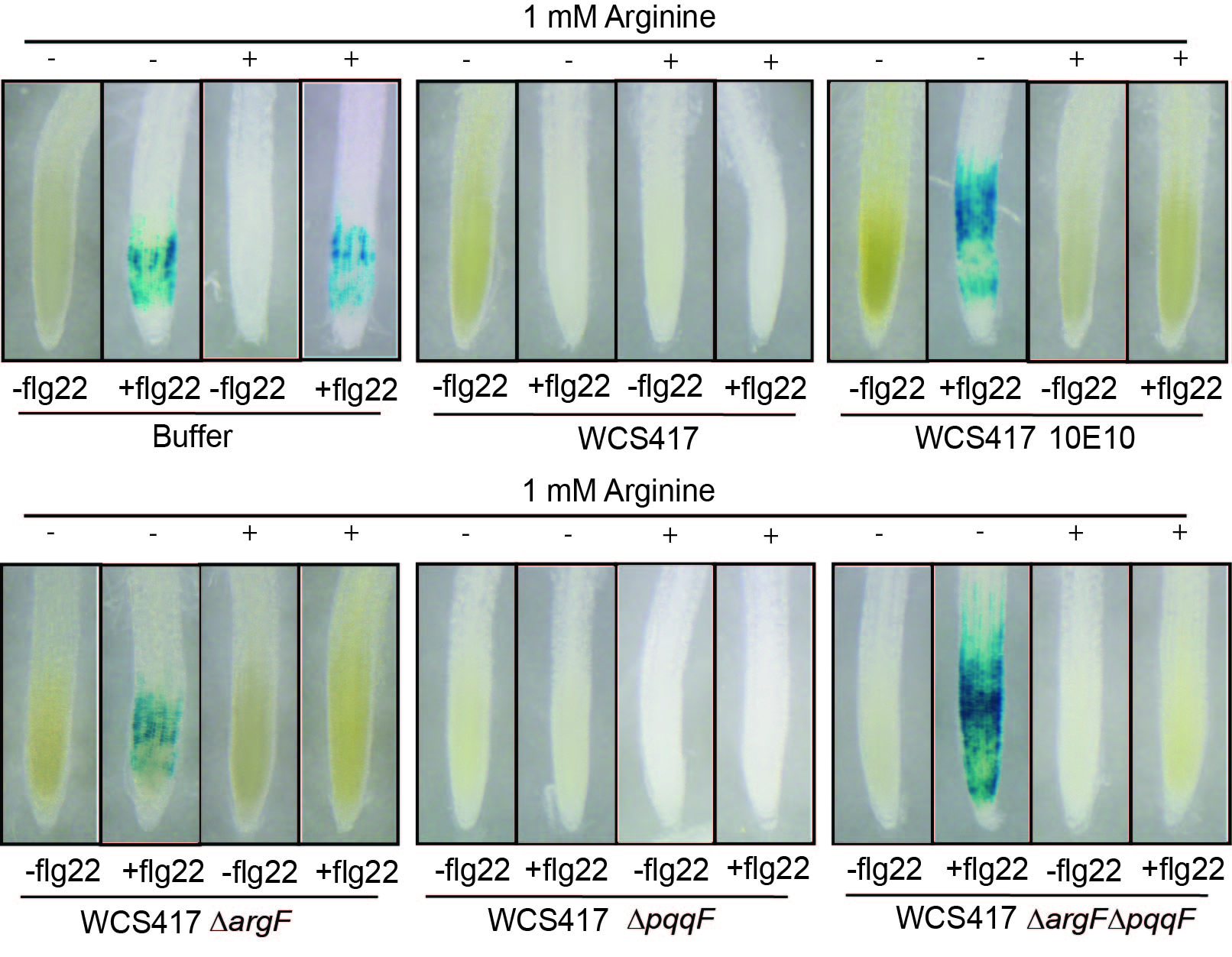

Supplement: FIG S3 [file mbio.03424-22-s0003.jpg]

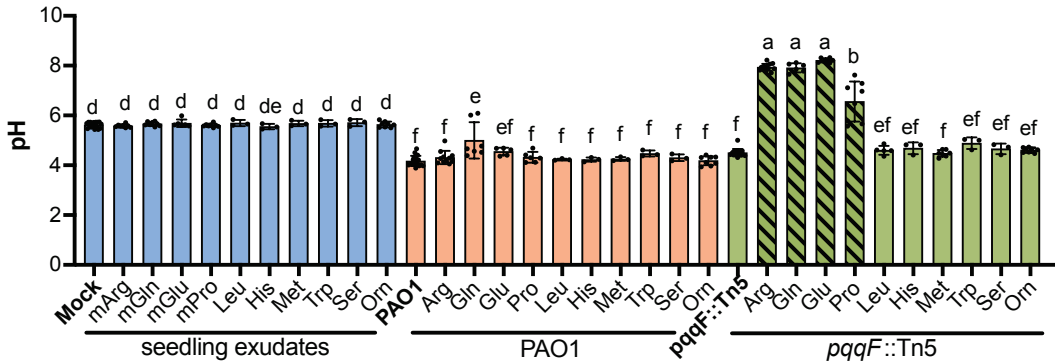

Supplement: FIG S4 [file mbio.03424-22-s0004.pdf]

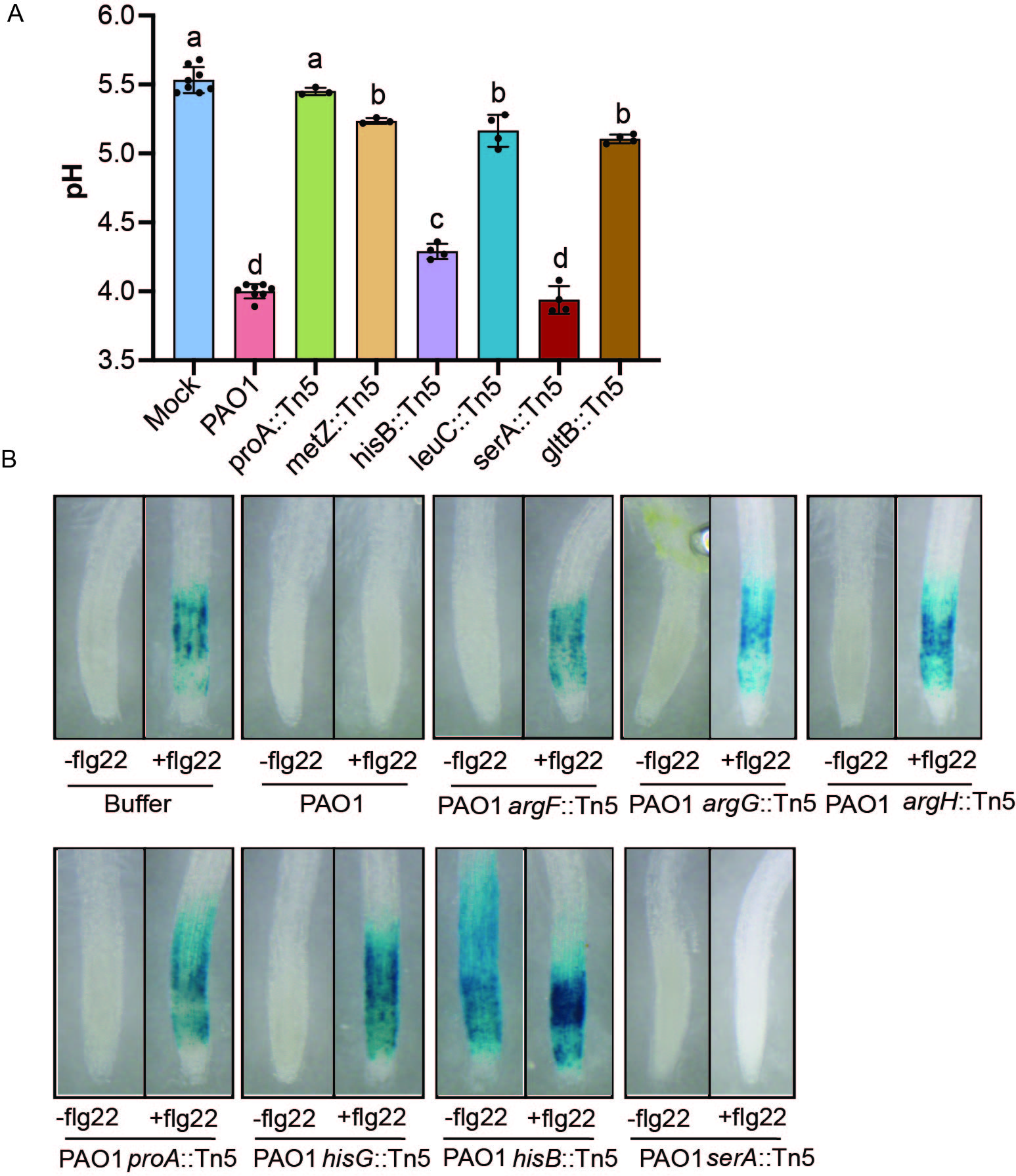

Supplement: FIG S5 [file mbio.03424-22-s0005.jpg]

OD<sub>600</sub>

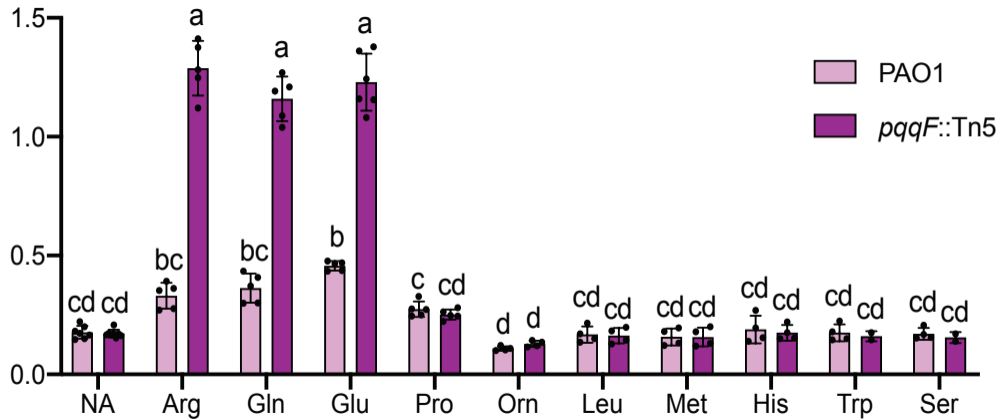

Supplement: FIG S6 [file mbio.03424-22-s0006.pdf]

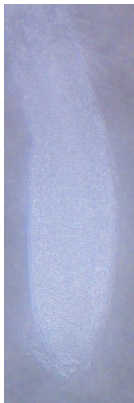

pH 5.7  
Buffer

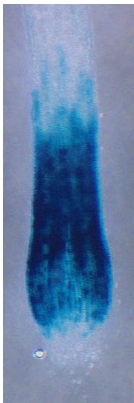

pH 5.7  
+flg22

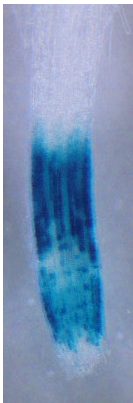

pH 8.0  
+flg22

Supplement: FIG S7 [file mbio.03424-22-s0007.pdf]

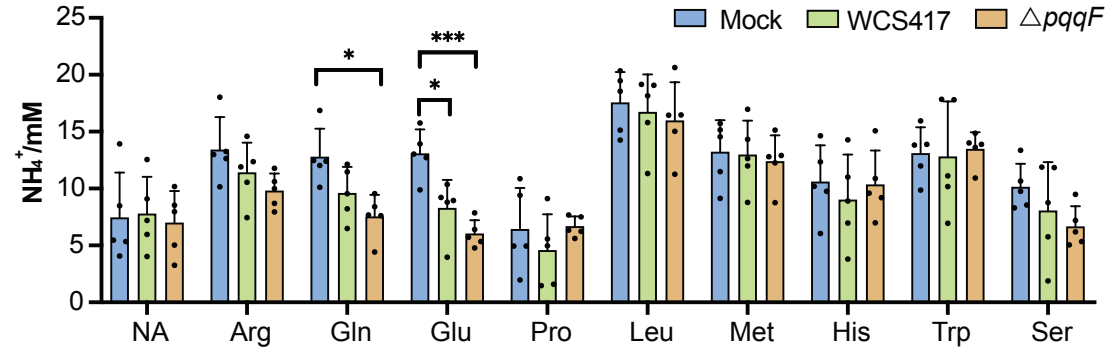

Supplement: FIG S8 [file mbio.03424-22-s0008.pdf]
